# Mitochondrial replacement in an iPSC model of Leber's hereditary optic neuropathy

**DOI:** 10.18632/aging.101231

**Published:** 2017-04-29

**Authors:** Raymond C.B. Wong, Shiang Y. Lim, Sandy S.C. Hung, Stacey Jackson, Shahnaz Khan, Nicole J. Van Bergen, Elisabeth De Smit, Helena H. Liang, Lisa S Kearns, Linda Clarke, David A. Mackey, Alex W. Hewitt, Ian A. Trounce, Alice Pébay

**Affiliations:** ^1^ Centre for Eye Research Australia, Royal Victorian Eye and Ear Hospital, East Melbourne, Australia; ^2^ Department of Surgery, Ophthalmology, the University of Melbourne, Melbourne, Australia; ^3^ O'Brien Institute Department, St Vincent's Institute of Medical Research, Victoria, Australia; ^4^ Centre for Ophthalmology and Vision Science, University of Western Australia, Lions Eye Institute, Nedlands, Australia; ^5^ School of Medicine, Menzies Institute for Medical Research, University of Tasmania, Hobart, Australia

**Keywords:** Leber's hereditary optic neuropathy, disease model, induced pluripotent stem cells, retinal ganglion cells, cybrid

## Abstract

Cybrid technology was used to replace Leber hereditary optic neuropathy (LHON) causing mitochondrial DNA (mtDNA) mutations from patient-specific fibroblasts with wildtype mtDNA, and mutation-free induced pluripotent stem cells (iPSCs) were generated subsequently. Retinal ganglion cell (RGC) differentiation demonstrates increased cell death in LHON-RGCs and can be rescued in cybrid corrected RGCs.

## INTRODUCTION

Patient somatic cells can be reprogrammed into iPSCs, by ectopic expression of a combination of reprogram-ming factors [1, 2]. These cells can subsequently be differentiated into cell types of interest, providing a platform for disease modeling, drug screening and gene therapy. In recent years, the field of iPSC disease modeling has focused on the use of isogenic control,* i.e.* by correcting the disease-causing mutation(s) to verify the disease phenotypes observed in the iPSC model. Gene editing technologies, such as TALEN or CRISPR/Cas9, emerged as tools to establish isogenic iPSC controls for nuclear gene mutations. However, the difficulty of genetically manipulating mtDNA represents an obstacle for generating iPSC isogenic controls to model mtDNA diseases. Mutations in mtDNA can be heteroplasmic (mutated mtDNA coexists with wild-type mtDNA) or homoplasmic (all mtDNA copies are mutated). iPSCs have been utilised to model mitochondrial diseases with mtDNA heteroplasmy [3]. Interestingly, mutated mtDNA heteroplasmy has been reported to segregate during iPSC reprogramming, similar to the mtDNA genetic bottleneck phenomenon observed during early embryogenesis. In Mitochondrial Encephalomyopathy Lactic Acidosis and Stroke-like episodes (MELAS) [4], Pearson Syndrome [5] and diabetes mellitus [6], iPSC reprogramming yielded both mutant mtDNA-rich iPSC clones and mutation-free iPSC clones. However, this approach would not be feasible for iPSC modeling of homoplasmic mtDNA disease, such as LHON.

LHON is characterized by loss of the RGCs [7]. In European populations, approximately 1 in 9,000 is a LHON carrier and it affects approximately 1 in 30,000 individuals, causing sudden visual loss predominantly [8]. All LHON cases are caused by mtDNA mutations that encode for the mitochondrial Complex I subunits [9, 10]. These homoplasmic mutations are shown to disrupt the activity of Complex I, leading to a decrease in bioenergetic production and an increased level of oxidative stress [11]. However, the precise mechanism for disease progression in LHON remains unknown. Here we report the use of iPSCs to model LHON, and demonstrate generation of isogenic iPSC controls by replacing LHON mtDNA using cybrid technology.

## RESULTS

**Figure 1:**
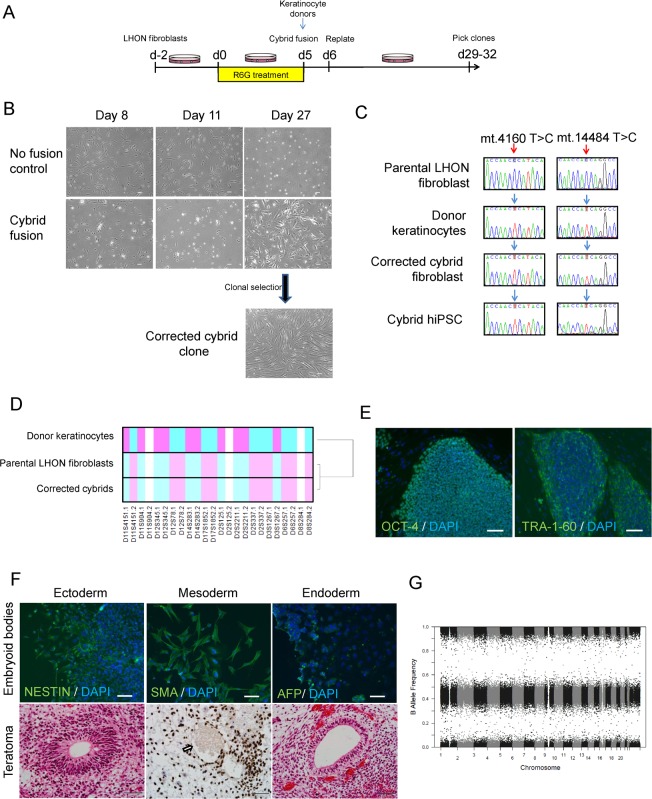
Using cybrid transfer to generate mutation-free LHON fibroblasts and iPSCs (**A**) Diagram of cybrid generation. Fibroblasts were pre-treated with the mitochondrial toxin rhodamine 6-G (R6G) then fused with healthy donor mitochondria obtained from normal keratinocytes. On day 29-32, proliferating colonies were picked and expanded.

We previously reported on the generation and characterisation of LHON iPSCs [12]. For this study, we utilised iPSCs from a healthy control (MRU11780), and a LHON patient (LHON Q1-4) with homoplasmic double mtDNA mutations m.4160T>C and m.14484T>C which affected the *MT-ND1* and *MT-ND6* genes respectively [12]. This LHON patient exhibited “LHON plus” phenotype, with clinical features including optic nerve atrophy, juvenile encephalopathy and peripheral neuropathy [13]. To generate an isogenic control for iPSC modeling, we utilised the cybrid technique to replace the mutant mtDNA in LHON fibroblasts. LHON fibroblasts were pre-treated with rhodamine 6-G to disable the transmission of endo-genous mtDNA, followed by fusion with donor mitochondria obtained from wild-type keratinocytes (Fig. 1A). After 27 days post-fusion, proliferating fibroblast colonies that are indicative of successful mitochondrial replacement were isolated and expanded (Fig. 1B). In contrast, no proliferating fibroblast colony was observed in control conditions that did not receive donor keratinocyte mitochondria (Fig. 1B). Out of 12 clones screened, we identified 1 cybrid clone with the corrected mtDNA genotype at m.4160 and m.14484 (Fig. 1C). Microsatellite analysis of a panel of 12 polymorphic markers confirmed that the corrected cybrid clone originated from the parental LHON fibroblasts, whereas the donor keratinocytes possessed a different microsatellite profile (Fig. 1D, Supplementary Table). We then generated iPSCs from the cybrid fibroblasts using the episomal method. Following reprogramming, three clones of cybrid iPSCs (CYB iPSC c1, CYB iPSC c2, CYB iPSC c3) were selected for this study. Importantly, all cybrid iPSC clones retained mtDNA correction with no detectable mutations at m.4160 and m.14484 (Fig. 1C, Supplementary Fig. 1). Further characterization demonstrated that the cybrid iPSCs expressed the pluripotent markers OCT-4 and TRA-1-60 (Fig. 1E, Supplementary Fig. 2). The derived cybrid iPSCs were also differentiated into cells of the three germ layers *in vitro* by embryoid body formation and *in vivo* by teratoma formation (Fig. 1F, Supplementary Figs. 2 and 3). Copy number variation analysis indicated no chromosomal abnormalities in the derived cybrid iPSC clones (Fig. 1G, Supplementary Fig. 4). Together, these results demonstrate the feasibility of using the cybrid technique to generate isogenic iPSC controls for mtDNA disease modeling.

**Figure 2:**
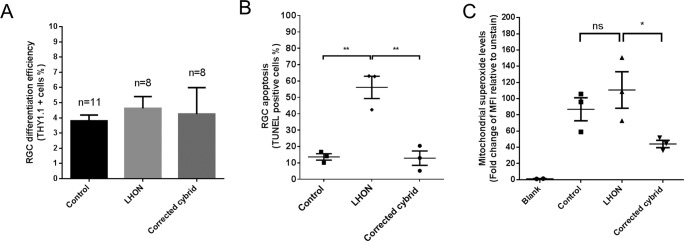
LHON disease phenotype in iPSC-derived RGCs can be reversed in corrected cybrid lines (**A**) Efficiency in RGC differentiation as assessed by the % of THY1.1 positive cells obtained post MACS sorting for control, LHON and corrected cybrid lines. Data are expressed as mean + SEM of independent samples (n=11 for control, n=8 for LHON and n=8 for corrected cybrid, expressed as pooled data of experimental repeats and biological repeats (3 clones)) (**B**) TUNEL assay revealed increased susceptibility to apoptosis in LHON RGCs and reversal in corrected cybrid lines. Data are expressed as mean of each clone, n = 3 clones, error bars = mean ± SEM. (**C**) Quantification of mitochondrial superoxide levels using MitoSOX in control, LHON and corrected cybrid RGCs. Error bars = ± SEM, n = 3 clones. Statistical significance was established by one way ANOVA followed by Dunnett's test for multiple comparisons, ** p<0.01, * p<0.05, ns: not significant.

Finally, we assessed the effect of the LHON mtDNA mutations in iPSC-derived RGCs. Control, LHON and cybrid iPSCs were directed to differentiate into RGCs by a stepwise differentiation method that we recently published, which demonstrated an enriched population of functional RGCs [14]. Three iPSC clones per patient were used and all clones were able to differentiate into RGCs with similar efficiency (Fig. 2A). Following RGC enrichment using MACS, TUNEL analysis revealed an increased level of apoptosis in LHON RGCs, from 13.6 ± 1.9% in control RGCs to 56.1 ± 6.8% in LHON RGCs (Fig. 2B). Importantly, this effect was reverted in the cybrid corrected RGCs, with the apoptosis level returned to control levels (Fig. 2B, 12.9 ± 4.4%), demonstrating that the increased susceptibility to cell death observed in LHON-RGCs is a direct consequence of LHON mtDNA mutations. Mitochondrial dysfunction in RGCs was then assessed using MitoSOX, which measures the levels of mitochondrial superoxide, as an indication of mitochondrial oxidative stress. Despite a trend of an increased superoxide levels in LHON RGCs compared to control RGCs, this difference in mitochondrial superoxide levels was not statistically significant, possibly due to high variations observed amongst the iPSC clones. Lower superoxide levels were also observed in corrected cybrid lines compared to LHON RGCs (Fig. 2C). Together these results suggest that the higher apoptosis observed in the LHON RGCs cannot be explained by elevated mitochondrial superoxide alone. Future studies to investigate other defects in LHON RGCs, such as ATP deficiency [15] or mitochondrial biogenesis [16], will help elucidate the mechanism underlying penetrance and disease progression of LHON.

## DISCUSSION

The recent development of mitochondrial replacement therapy using pronuclear transfer offers exciting prospects to correct mtDNA mutations in the early human embryo [17]. However, pronuclear transfer is technically challenging, especially in cells of small size, and requires specialised equipment. Here, we describe for the first time a cybrid approach to correct mtDNA mutations in an iPSC disease model. Compared to pronuclear transfer, the cybrid technique is easier to perform and can be adapted to generate an isogenic control for iPSC models of homoplasmic mtDNA diseases. Reduction of mutant mtDNA load, rather than complete replacement of mutant mtDNA with wild-type mtDNA, might be sufficient for phenotypic rescue in iPSC isogenic controls. In support of this, previous studies have shown that selective elimination of mutant mtDNA by mitochondria-targeted TALEN can reduce the mutant mtDNA loads and restore the mitochondrial dysfunction [18]. Very recently, zinc fingers were also used for the successful replacement of heteroplasmic mtDNA [19]. Our results show an increased suscep-tibility to apoptosis in LHON iPSC-derived RGCs. Importantly, apoptosis was returned to normal levels in the cybrid-corrected RGCs, hence demonstrating that the increased susceptibility to cell death observed in RGCs is a direct consequence of LHON mtDNA mutations.

In summary, our approach shows the advantage of using LHON patient-derived iPSCs and their isogenic cybrid control as a platform for the study of the fundamental mechanisms underlying LHON pathogenesis. More-over, the cybrid technique provides a feasible strategy to correct mtDNA mutations in iPSC models, which could be applied to model other mtDNA diseases.

## MATERIALS AND METHODS

### Ethics

All experimental work performed in this study was approved by the Human Research Ethics Committees of the Royal Victorian Eye and Ear Hospital (11/1031H) and the University of Melbourne (0605017) [20,21], and with the Animal Ethics Committee of St Vincent's Hospital (002/14), in accordance with the requirements of the National Health & Medical Research Council of Australia and conformed with the Declarations of Helsinki.

### Cell culture

Cell lines used in this study consisted of control (MRU11780 [12]), LHON (LHON Q1-4 carrying m.14484 & m.4160 mtDNA mutations [12]) and cybrid-corrected lines. Fibroblasts were cultured in DMEM medium supplemented with 10% fetal calf serum, 1 x L-glutamine, 0.1 mM non-essential amino acids and 0.5× penicillin/streptomycin (all from Invitrogen). iPSC lines were cultured on mitotically inactivated mouse embryonic fibroblasts feeders in the presence of DMEM/F-12 medium supplemented with 1 x GlutaMAX, 20% knockout serum replacement, 10 ng/ml basic fibroblast growth factor, 0.1 mM non-essential amino acids, 100 μM β-mercaptoethanol and penicillin/streptomycin (all from Invitrogen) and passaged weekly as previously described [12].

### Cybrid generation

Cybrid transfer was performed as described [22,23]. Briefly, human epidermal keratinocytes (System Bioscience) were used as donor by enucleation using cytochalasin B treatment and high speed centrifugation. LHON Q1-4 fibroblasts were pre-treated with 2.5μg/ml rhodamine 6-G for 5 days, before fusion with donor cell cytoplasts using polyethylene glycol. On the next day, fused cells were replated and allowed to culture for up to 32 days. Proliferating colonies were picked and expanded using cloning cylinders (Corning). Isolated fibroblast clones were genotyped for mtDNA mutations at mt.4160 and mt.14484 to screen for successful mitochondrial replacement.

### Microsatellite analysis

Microsatellite analysis was performed by the Australian Genome Research Facility, using 12 polymorphic markers from the Applied Biosystems linkage mapping set. The markers utilised are D2S2211, D2S125, D2S337, D3S1267, D6S257, D8S284, D11S904, D11S4151, D12S78, D12S345, D14S283 and D17S1852. Hierarchical clustering and heatmap is generated using R.

### iPSC generation and characterisation

Reprogramming of cybrid-corrected fibroblasts was performed as described in [24]. Episomal vectors expressing OCT4, SOX2, KLF4, L-MYC, LIN28 and shRNA against p53 were gifts from Shinya Yamanaka (Addgene #27077, 27078, 27080). *In vitro* differentiation of iPSCs was performed by embryoid bodies and characterised as described in [12]. *In vivo* teratoma assay was performed by transplanting iPSCs into a vascularized tissue engineering chamber in immuno-deficient rats, as described in [24]. Histological analysis was performed on the teratoma samples after 4 weeks. Copy number variation (CNV) analysis of original fibroblasts and iPSCs was performed using Illumina HumanCore Beadchip arrays. CNV analyses were performed using PennCNV with default parameter settings [25]. Chromosomal aberrations were deemed to involve at least 10 contiguous single nucleotide polymorphisms (SNPs) or a genomic region spanning at least 1MB [25,26].

### RGC differentiation

iPSCs were directed for retinal differentiation and enriched for RGCs using MACS THY1.1 microbeads (Miltenyi Biotech) as described in our previous study [14]. On day 30, RGC differentiation efficiency is determined as described previously [14].

### Immunochemistry

Standard immunochemistry procedure was performed using mouse anti-OCT3/4 (#SC-5279, Santa Cruz Biotechnology), mouse anti-TRA-1-60 (#MAB4360, Millipore), mouse anti-NESTIN (#AB22035, Abcam), mouse anti-alpha-fetoprotein (AFP, #ST1673, Millipore), mouse anti-smooth muscle actin (SMA, #MAB1420, R&D systems). Cells were then immuno-stained with the appropriate conjugated secondary antibodies (Alexa Fluor 488, Molecular probes-Invitrogen). Nuclei were counter-stained with DAPI (Invitrogen). Immunohistochemistry for human cells was performed using rabbit anti-Ku80 (#AB80592, Abcam) followed by biotinylated goat anti-rabbit secondary antibody (Vector Laboratories) and avidin-biotinylated-peroxidase complex (Vector Laboratories). Peroxidase activity was visualised with diamino-benzidine chromogen (Dako) and sections were counterstained with hematoxylin. Specificity of the staining was verified by the absence of staining in negative controls consisting of the appropriate negative control immunoglobulin fraction.

### Mitochondrial superoxide measurement

Quantification of mitochondrial superoxide was performed on day 30 iPSC-derived RGCs using MitoSOX (Invitrogen) according to the manufacturer's instructions. Briefly, trypsinized cells were stained with Mitosox for 10 minutes at 37°C. Subsequently, samples were processed by flow cytometry using the MACSquant (Miltenyi). The median fluorescence intensity was determined using the MACSQuantify software and normalized to unstained control. For each clone, a minimum of 83,000 cells were processed by FACS.

### Apoptosis assay

TUNEL assay was performed on day 37-41 iPSC-derived RGCs using the *In situ* Cell death detection kit (Roche) following manufacturer's instructions. Floating apoptotic bodies were collected and the enriched RGCs were harvested by trypsinization. Quantitation of TUNEL positive cells was measured by flow cytometry using the MACSquant (Miltenyi). Apoptosis was assessed in basal conditions, without addition of any stressor or inducer of apoptosis. Generally, a minimum of 10,000 cells per clone were processed by FACS. In clones where <10,000 cells were obtained, experimental repeats were performed to obtain >10,000 cells.

### Statistical analysis

All statistical analyses and graphical data were generated using Graphpad Prism software (v5.04, www.graphpad.com) or in the R statistical environment (v3.1.2, https://cran.r-project.org/). Statistical methods utilised were One-way ANOVA followed Dunnett's multiple comparisons test for multiple comparison. Statistical significance was established as * p<0.05 and **p<0.01.

## SUPPLEMENTARY MATERIAL TABLE AND FIGURES



## Abbreviations

cyb: Cybrid
iPSCs: induced pluripotent stem cells
LHON: Leber hereditary optic neuropathy
MELAS: Mitochondrial Encephalomyopathy Lactic Acidosis and Stroke-like episodes
mt: mitochondrial
RGCs: retinal ganglion cells
