# Supplementary material for: Mitochondrial replacement in an iPSC model of Leber's hereditary optic neuropathy
Source: Aging (Albany NY). 2017 Apr 29;9(4):1341–7. doi: 10.18632/aging.101231 (PMC5425131; doi:10.18632/aging.101231)
Supplement: Supplementary file 1 [file aging-09-1341-s001.pdf]

SUPPLEMENTARY MATERIAL

Supplementary Table 1. Profiles of 12 microsatellite markers for donor keratinocytes, parental LHON fibroblasts (LHON Q1-4) and corresponding corrected cybrids.

Marker	Sample	Allele 1	Allele 2
D11S4151	Donor keratinocytes	336	340
	Parental LHON fibroblasts	334	342
	Corrected cybrids	334	342
D11S904	Donor keratinocytes	191	201
	Parental LHON fibroblasts	187	201
	Corrected cybrids	187	201
D12S345	Donor keratinocytes	217	237
	Parental LHON fibroblasts	215	235
	Corrected cybrids	215	235
D12S78	Donor keratinocytes	188	190
	Parental LHON fibroblasts	194	196
	Corrected cybrids	194	196
D14S283	Donor keratinocytes	141	151
	Parental LHON fibroblasts	135	149
	Corrected cybrids	135	149
D17S1852	Donor keratinocytes	303	305
	Parental LHON fibroblasts	309	309
	Corrected cybrids	309	309
D2S125	Donor keratinocytes	94	96
	Parental LHON fibroblasts	92	96
	Corrected cybrids	92	96
D2S2211	Donor keratinocytes	252	252
	Parental LHON fibroblasts	246	248
	Corrected cybrids	246	248
D2S337	Donor keratinocytes	294	296
	Parental LHON fibroblasts	298	308
	Corrected cybrids	298	308
D3S1267	Donor keratinocytes	99	121
	Parental LHON fibroblasts	113	117
	Corrected cybrids	113	117
D6S257	Donor keratinocytes	171	179
	Parental LHON fibroblasts	175	185
	Corrected cybrids	175	185
D8S284	Donor keratinocytes	273	285
	Parental LHON fibroblasts	273	295
	Corrected cybrids	273	295

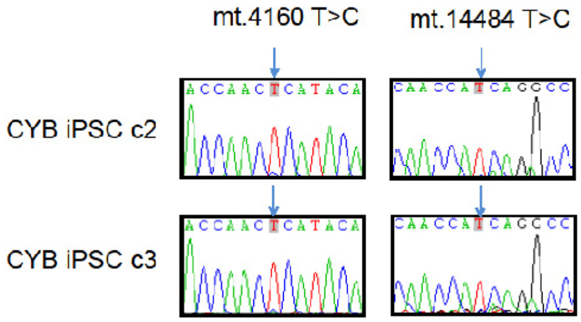

Supplementary Figure 1. Genotyping confirming two cybrid clones (CYB iPSC c2, CYB iPSC c3) with corrected mtDNA. Blue arrows indicate lack ok LHON mutations at m.4160T>C and m.14484T>C.

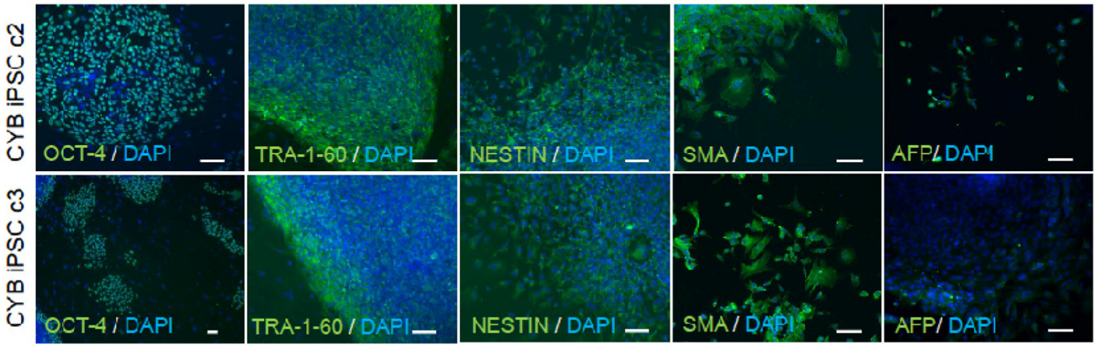

Supplementary Figure 2. Characterization of two cybrid iPSCs (CYB iPSC c2, CYB iPSC c3). Immunostaining showed expression of the pluripotency markers OCT-4 and TRA-1-60 in cybrid iPSCs. Differentiation of cybrid iPSCs by embryoid body formation with cells positive for NESTIN (ectoderm), SMA (mesoderm) and AFP (endoderm). Cells were counterstained with DAPI (blue). Scale bars = 100 µm.

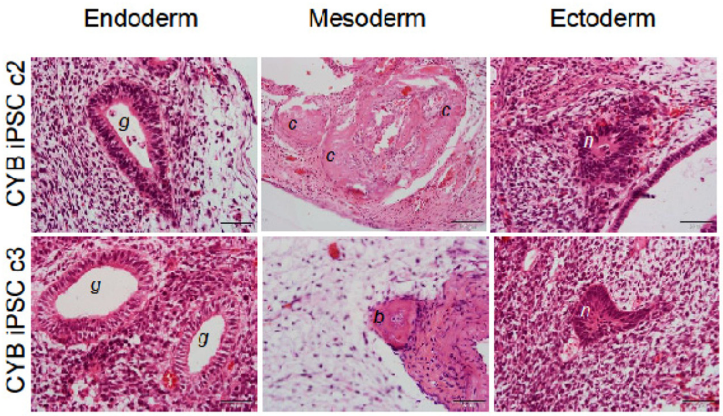

Supplementary Figure 3. Teratoma formation upon transplantation of cybrid iPSCs (CYB iPSC c2, CYB iPSC c3) in nude rats, showing differentiation to endoderm, mesoderm and ectoderm. G: gut-like epithelium; c: cartilaginous structure; b: bone-like structure; n: neural rosette. Scale bars: 50 µm.

A

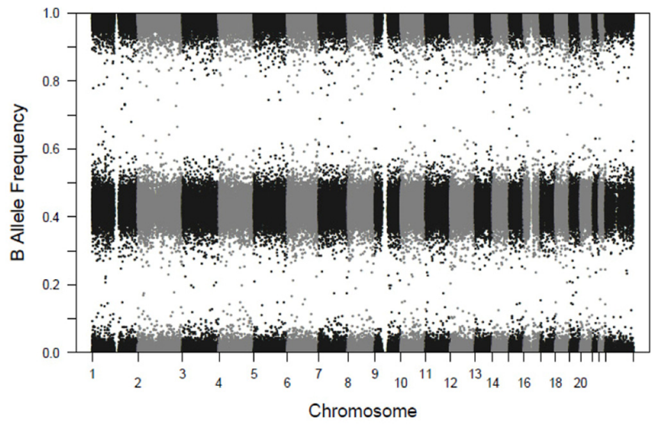

B

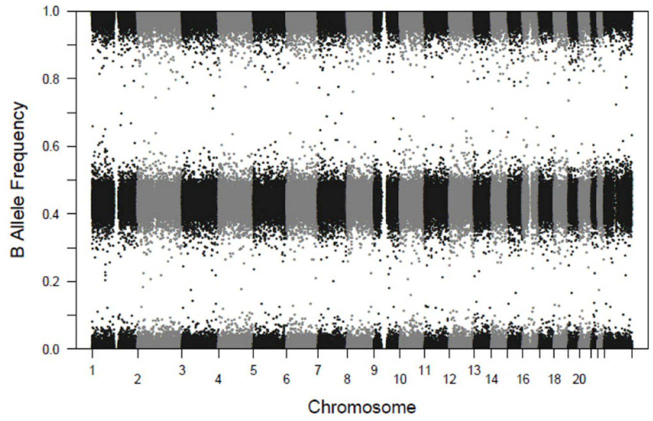

Supplementary Figure 4. Copy number variation analysis showing normal karyotype in two cybrid iPSC clones, (**A**) CYB iPSC c2; (**B**) CYB iPSC c3.
